# Temporal Characteristics of High-Frequency Lower-Limb Oscillation during Freezing of Gait in Parkinson's Disease

**DOI:** 10.1155/2014/606427

**Published:** 2014-07-02

**Authors:** Don A. Yungher, Tiffany R. Morris, Valentina Dilda, James M. Shine, Sharon L. Naismith, Simon J. G. Lewis, Steven T. Moore

**Affiliations:** ^1^Human Aerospace Laboratory, Department of Neurology, Mount Sinai School of Medicine, Box 1052, 1 Gustave L. Levy Place, New York, NY 10029, USA; ^2^Parkinson's Disease Research Clinic, Brain and Mind Research Institute, University of Sydney, Sydney, NSW 2006, Australia; ^3^Robert and John M. Bendheim Parkinson and Movement Disorders Center, Department of Neurology, Mount Sinai School of Medicine, New York, NY 10029, USA

## Abstract

A cardinal feature of freezing of gait (FOG) is high frequency (3–8 Hz) oscillation of the legs, and this study aimed to quantify the temporal pattern of lower-body motion prior to and during FOG. Acceleration data was obtained from sensors attached to the back, thighs, shanks, and feet in 14 Parkinson's disease patients performing timed-up-and-go tasks, and clinical assessment of FOG was performed by two experienced raters from video. A total of 23 isolated FOG events, defined as occurring at least 5 s after gait initiation and with no preceding FOG, were identified from the clinical ratings. The corresponding accelerometer records were analyzed within a 4 s window centered at the clinical onset of freezing. FOG-related high-frequency oscillation (an increase in power in the 3–8 Hz band >3 SD from baseline) followed a distal to proximal onset pattern, appearing at the feet, shanks, thighs, and then back over a period of 250 ms. Peak power tended to decrease as the focus of oscillation moved from feet to back. There was a consistent delay (mean 872 ms) between the onset of high frequency oscillation at the feet and clinical onset of FOG. We infer that FOG is characterized by high frequency oscillation at the feet, which progresses proximally and is mechanically damped at the torso.

## 1. Introduction

Freezing of gait (FOG), a paroxysmal block of movement associated with gait initiation, turning, or negotiating an obstacle, is often described by the Parkinson's disease (PD) patient as the sensation that their feet are “stuck to the ground.” FOG is generally regarded as a late feature of PD associated with disease duration and severity [1, 2]; in a recent survey of 6620 PD patients 80% of respondents in the late stages of the disease reported frequent freezing of gait [3]. However, some early-stage PD patients (up to 26% of patients not yet administered levodopa [2, 4, 5] and 10% with mild symptoms [3]) also experience FOG. The increased risk of falling associated with freezing has a devastating impact on patients' well-being [1]; loss of mobility and independence significantly impair quality of life [6], and fall-related injuries such as hip fractures [7] are associated with a high morbidity and mortality [8]. FOG-related gait impairment increases the likelihood of nursing home admission [1, 9], imposing a considerable economic burden on the health care system [10].

The underlying pathophysiology of FOG is unknown, and evaluation and treatment are often suboptimal [1, 11]. Although freezing has been reported in drug-naive individuals [2, 4, 5], there is an increased prevalence in advanced disease and in the clinical “off” state, highlighting the key role of striatal dopamine depletion in its pathogenesis [4]. Increasing levodopa dosage can alleviate “off” state freezing [1] (whereas dopamine agonist treatment has been associated with an increased frequency of FOG in early PD [12]). However, FOG commonly shows only partial response to levodopa [13] and can also be observed in the “on” (medicated) state [14], suggesting that the pathophysiology of freezing is not solely related to striatal dopamine levels. Selegiline, a monoamine oxidase type B inhibitor, reduced the development of FOG in early PD in a prospective double-blind trial [4], but treatment of levodopa-resistant FOG with selegiline in clinical practice has shown little success [1]. Patients may undergo deep brain stimulation surgery to relieve symptoms of freezing, with electrodes implanted in either the subthalamic (STN) or pedunculopontine (PPN) nuclei [15–17]. Such surgical interventions are currently viewed as a treatment option only in the latter stages of Parkinson's disease and correct patient selection is critical for success [18]; of particular concern is the fact that several years after an initially beneficial response some patients may develop severe intractable gait and balance problems [1].

FOG is considerably more complicated than the sudden cessation of forward motion. A number of subtypes have been identified [19]: difficulty initiating gait (start hesitation), freezing during linear locomotion (runway FOG), hesitation upon target approach, and turning. Several lines of evidence suggest that pathologic gait precedes the motor block by several steps, with sequentially shorter step lengths [20] and abnormal timing of tibialis anterior and gastrocnemius muscle activation prior to freezing [21]. In addition, FOG severity is associated with increased cognitive and limbic loads. Impaired set-shifting (trail making test) performance was correlated with the number and duration of freezing episodes during walking in the “off” state [22]. The timed up and go test, which consists of standing from a seated position, walking 5 m to a target box marked on the floor, turning 180° (or more), and returning to the chair and sitting down, is an effective means for eliciting FOG in the clinic [23, 24].

We have demonstrated that FOG is associated with the appearance of high-frequency (3–8 Hz) components in foot, shank, thigh, and lumbar acceleration [23–26]. This high frequency pattern is apparent clinically in the lower limb “trembling in place” observed during and after the motor block, although lower limb oscillations in the 3–8 Hz range can be subclinical [23]. The aim of this study was to quantify the temporal pattern of onset of high-frequency leg oscillation during freezing using sensors mounted on the lumbar back, thighs, shanks, and feet and relate this to the clinical onset of a freeze event as determined from video observation. A better understanding of the temporal relationship between the onset of trembling in the lower limbs may provide insight into the central underlying cause of FOG, as well as having potential clinical application as an early detection system to warn patients of an imminent gait arrest.

## 2. Methods

### 2.1. Patients

Fourteen PD patients (9 male and 5 female) with a clinical history of FOG were recruited from the Parkinson's Disease Research Clinic at the Brain and Mind Research Institute, University of Sydney. These patients were part of a larger group of 24 participants in a previously published study [24] and were selected based on the presence of “isolated” FOG events observed in* post hoc* analysis as described below. All patients satisfied UKPDS Brain Bank criteria, had a Mini-Mental State Examination (MMSE) score of ≥24, and were deemed unlikely to have dementia or major depression according to DSM-IV criteria by consensus rating of a neurologist (SJGL) and a neuropsychologist (SLN). Participants were assessed in the practically-defined “off” state following overnight withdrawal of dopaminergic therapy. Patient characteristics were a mean age of 70.8 years [SD 9.4], disease duration 10.1 years [SD 9.6], Hoehn and Yahr stage 2.7 [SD 0.6], and UPDRS-Section III 40.7 [SD 12.4]. None of the patients described any increase in freezing behavior following the administration of their usual dopaminergic therapy. All subjects gave written, informed consent according to the Declaration of Helsinki and the study was approved by the University of Sydney's Institutional Review Board.

### 2.2. Locomotor Task

The 14 patients performed a total of 81 timed up-and-go (TUG) tasks (mean 5.8 [SD 1.8] per subject) to provoke FOG on a standardized 5-m course [23–25, 27]. Participants, starting from a seated position, walked 5 m to a 0.6 × 0.6 m box marked on the floor with yellow tape, in which a turn to the left or right was performed before returning to the chair. Walking trials were recorded on a digital video camera from a consistent vantage point for later analysis, and each video showed a complete TUG trial starting and ending in the seated position.

### 2.3. Data Acquisition

**Figure 1:**
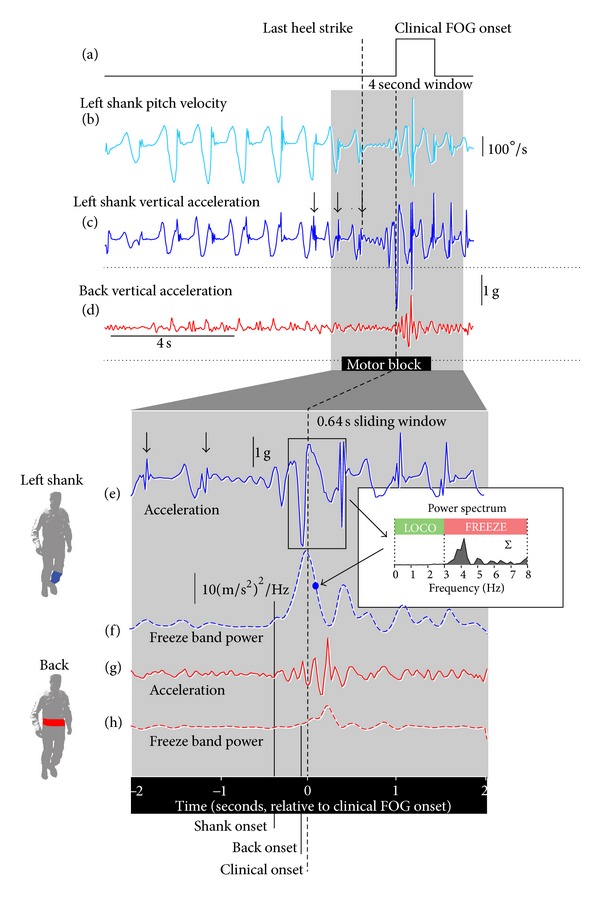
Typical FOG event. (a) Clinicians rating of FOG from video generated a binary trace, with “1” indicating a freeze event. (b) Left shank pitch velocity. (c) Left shank vertical acceleration. (d) Back vertical acceleration. (e) 4 s of shank acceleration, centered on the clinical onset of FOG. (f) Freeze band power of the shank was calculated at each point in time within a 0.64 s window by summation of power in the 3–8 Hz band. (g) 4 s of back acceleration, centered on the clinical onset of FOG. (h) Freeze band power of the back was calculated at each point in time within a 0.64 s window by summation of power in the 3–8 Hz band. In this instance, the onset of freeze band power (>3 SD above baseline) occurred initially in the shank, approximately 300 ms prior to onset at the back.

Subjects were instrumented with seven inertial measurement units (IMUs — Xsens MTx, Enschede, Netherlands) firmly secured to the lumbar region of the back at approximately L2, the lateral aspect of each thigh and shank, and the superior aspect of each midfoot, with elasticized straps. The IMUs were small (38 × 53 × 21 mm; 30 g) and did not interfere with natural movement. During testing each IMU acquired triaxial linear acceleration, angular velocity, and readings of the Earth's magnetic field, transmitted wirelessly to a computer at a sample rate of 50 Hz (only longitudinal [vertical] acceleration of each body segment was considered in this study—see Figures 1(c), 1(d), 1(e), and 1(g)). Synchronization of the video and accelerometer recordings was performed prior to data collection by alignment of the video camera and data-acquisition computer clocks.

### 2.4. Clinical Assessment of FOG

The clinical onset of freezing episodes were determined for each video utilizing a FOG tagging program described previously [23, 24]. Two clinicians experienced in FOG (JMS and SJGL) used their best clinical judgment to identify FOG episodes from video, tagging the onset of a freeze by pressing the “T” key and holding down the key for the duration of each event. Video editing tools enabled the raters to precisely adjust the onset and offset of FOG. When satisfied with the result, clinical ratings were saved as a binary signal at the video frame rate (30 Hz), with a zero baseline and 1 indicating freezing of gait (see Figure 1(a)). The results from the two raters were combined using a logical OR operation, resulting in a single binary FOG waveform for each TUG task (Figure 1(a)). This rater pairing achieved high agreement (intraclass correlation coefficient [ICC] for number of FOG = 0.82; percent time frozen ICC = 0.99) in a previous study using this scoring technique [24].

### 2.5. Data Analysis

The clinical raters identified 23 trials as having an “isolated” freeze, defined as a FOG event occurring no earlier than 5 seconds following gait initiation with no preceding FOG. This ensured that each freeze episode analyzed was “clean,” with no residual “trembling” from a prior freeze that could compromise the determination of FOG onset. Four seconds of acceleration data from each of the seven sensors, centered on the clinically-defined onset of freezing (Figure 1(a)), were analyzed for each FOG episode. A time-frequency analysis was conducted using the Gabor spectrogram function in Labview (National Instruments, Austin, Texas). At each point in time (20 ms intervals) a spectrogram was calculated within a window of 0.64 s, and the power of frequency components between 3 and 8 Hz summed (Figures 1(e) and 1(f)). This measure of “freeze” band power has previously been demonstrated as a robust objective marker of FOG [23, 24, 26], which is characterized by the development of significant power in the 3–8 Hz band and a diminishing of power for frequencies below 3 Hz (the “locomotor” band). High frequency (3–8 Hz) lower limb oscillation was observed in all 23 FOG episodes studied. A baseline of freeze band cumulative power was calculated from the first 5 s of gait prior to FOG, and the onset of freeze-related high frequency (3–8 Hz) acceleration was defined as the point in time where freeze band power increased >3 SD from this baseline (Figures 1(f) and 1(h)). Differences in the onset of 3–8 Hz oscillation in the thighs, shanks, and feet, and peak power were tested for significance using ANOVA, with alpha values set at 0.05. In addition, the time between the last heel strike (left or right) prior to the motor block and the clinical onset of FOG (Figure 1(a)) was determined for each freeze event.

## 3. Results

Data from a FOG event illustrates the pattern of high frequency accelerations observed prior to and during freezing (Figure 1). The heel strike prior to cessation of forward motion occurred at the left foot (Figures 1(b) and 1(c)), with clinical onset of FOG occurring 850 ms later (Figure 1(a)). Following the last heel strike a small, high frequency oscillation of the shank was observed (Figure 1(e)) which greatly increased in magnitude approximately 250 ms before the clinical onset of FOG. Indicators of the impending freeze event are evident several strides before the motor block. Left shank swing phase velocity was successively lower for the three strides prior to FOG (Figure 1(b)), and high frequency bursts of acceleration are evident at the final three heel strikes of shank acceleration (Figure 1(c); black arrows), which continue for several steps after the resumption of forward motion. (These high frequency bursts are reflected in the freeze band power (Figure 1(f)) as early as 2 s prior to clinical FOG onset but were just below the onset threshold). Onset of FOG-related high frequency acceleration was determined by summation of the power in the freeze band (3–8 Hz) over the 4 s period centered on clinical FOG onset. In this example, shank freeze band power reached the onset threshold (>3 SD above baseline) 375 ms prior to clinical FOG onset. Back freeze band power reached the onset threshold approximately 300 ms later.

**Figure 2:**
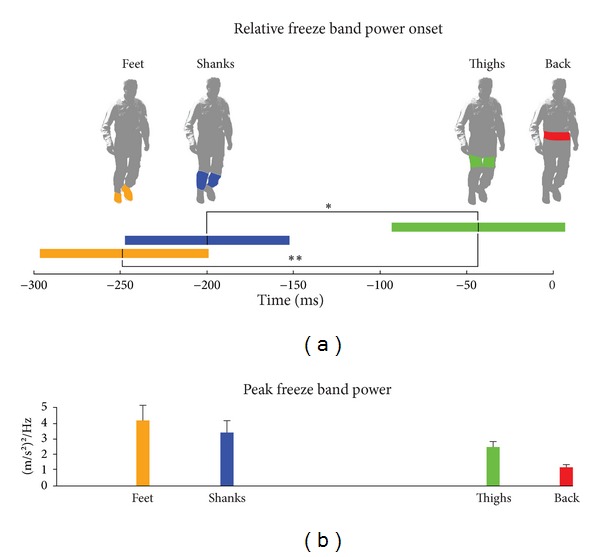
(a) Onset of lower limb freeze band activity relative to the back sensor (mean and SEM of both limbs). High frequency (3–8 Hz) oscillation occurred first in the feet, shanks, thighs, and then back, over a period of 250 ms. (b) Peak power (mean and SEM) decreased from the distal to proximal sensors.

Onset of FOG-related high frequency oscillations followed a distal to proximal (feet to back) progression. Relative to the back sensor, the onset of freeze band power (>3 SD above baseline) occurred first at the feet (left −232 ms [SEM 43]; right −259 ms [SEM 45]), then the shanks (left −226 ms [SEM 41]; right −194 ms [SEM 45]) and thighs (left −66 ms [SEM 43]; right −16 ms [SEM 43]). ANOVA for onset times yielded statistical significance for both left (*P* = 0.006) and right (*P* = 0.016) limbs. As no significant differences were observed between left and right onset the results were combined for further analysis (Figure 2(a): feet: 250 ms [SEM 48]; shanks: 210 ms [SEM 50]; thighs: 41 ms [SEM 51]; all times relative to back onset). ANOVA for bilateral onset times yielded statistical significance (*P* = 0.01). The onset of high frequency oscillation at the feet was significantly earlier than at the thighs (t[45] = 2.61, *P* = 0.006); similarly, oscillation appeared at the shanks before the thighs (t[45] = 2.12, *P* = 0.02). The difference in onset time for the feet and shanks was not significant. Peak freeze band power decreased from distal to proximal sensors (Figure 2(b)) but did not reach statistical significance, (*P* = 0.09). There was a significant delay of 872 ms [SEM 191] between freeze band power onset at the feet and the clinical onset of FOG as determined from video observation (t[45] = −4.29, *P* < 0.001). Clinical FOG onset lagged final heel strike by 496 ms [SEM 113]. The difference between the timing of high frequency oscillation onset at the feet and final heel strike was at the threshold of significance (t[45] = 1.69, *P* = 0.049); thus, on average, high frequency acceleration was detected in the lower limbs prior to motion cessation.

**Figure 3:**
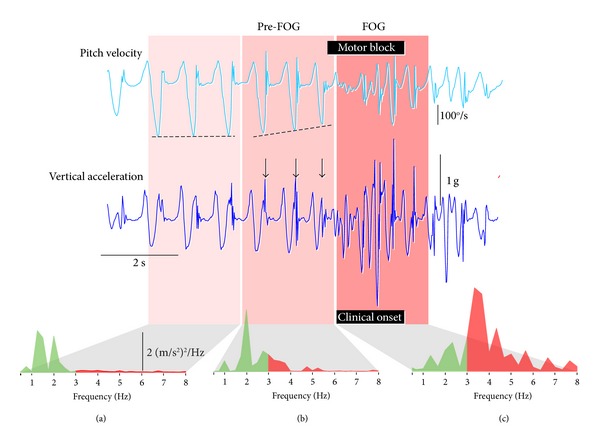
Development of high frequency lower limb oscillation prior to and during FOG. (a) Four to six strides prior to freezing, stride (peak swing phase velocity) was consistent and little power was observed in the 3–8 Hz band. (b) In the three strides prior to FOG, peak swing phase velocity (and therefore stride length) successively decreased and high frequency oscillations developed at heel strike, reflected in growing power in the 3–8 Hz band. (c) During FOG, forward progression had ceased and high frequency oscillations dominated the frequency spectrum.

This phenomenon can be observed in a FOG event from another participant (Figure 3). Six strides (left leg) can be observed prior to the motor block. Strides 1 to 3 (Figure 3(a)) exhibit a consistent swing phase velocity and a frequency analysis shows power concentrated in the locomotor range of 1-2 Hz. In the three strides prior to FOG (Figure 3(b)) the peak swing phase velocity steadily decreases, indicative of sequentially smaller strides, and high frequency acceleration bursts are apparent at heel strike (Figure 3(b) black arrows), as reflected in the significant power developing in the 3–8 Hz band of the frequency spectrum. Clinical onset of FOG is associated with the breakdown of gait and the high frequency acceleration power is considerably greater than power in the locomotor (<3 Hz) band (Figure 3(c)).

## 4. Discussion

The results of this study demonstrate a distal to proximal (feet to back) pattern of onset of high-frequency (3–8 Hz) oscillations in the lower body during freezing, which precedes the clinically observed onset of FOG. In addition, peak power tended to decrease from distal to proximal body segments. The temporal pattern of freeze band onset and distal-to-proximal power attenuation suggest that the characteristic “trembling” of FOG is driven by high-frequency behavior at the feet and ankles, which is increasingly damped in a bottom-up manner, perhaps due to the greater mass of the torso.

Our findings suggest that high frequency oscillation intrudes upon lower limb motion prior to the cessation of motion, consistent with previous reports of abnormal rhythmicity in tibialis anterior and gastrocnemius muscle activation [21] and successively smaller stride length [20] in the steps preceding FOG. This suggests that FOG-related high frequency lower limb acceleration at the feet is not, at least initially, an attempt to overcome a motor block, but part of a pathologic pattern of gait that precedes a freeze event by up to three strides.

Although the pathophysiological basis of FOG is not clear, these results, and those of prior studies [21, 23, 24, 26, 28, 29], suggest that a cardinal clinical feature of freezing is the development of high-frequency oscillations in the lower limbs of central origin. A recent model [30] has proposed that overactive and pathologically synchronous firing in the globus pallidus interna (GPi) (with subsequent inhibition of the pedunculopontine nuclei and thalamus) underlies FOG, and in a “Parkinsonian” state GPi neurons fire bursts of action potentials within a range (3–8 Hz) [31] corresponding to the freeze band [23, 26].

The primary limitations of this study are the relatively limited number of patients and FOG events studied, and the post-hoc nature of the analysis. Although care was taken to select “clean” freezing episodes with no preceding FOG, we cannot discern whether freezing was of the “runway” or “target hesitation” subtype, and “start hesitation” was not analyzed due to a lack of baseline data necessary for determining a threshold for FOG onset. Another issue, in common with the vast majority of published FOG studies, is the variability in clinical scoring of FOG. In this study observed FOG onset lagged the last heel strike by half a second, which is of little concern when clinically evaluating a patient but insufficiently accurate when mapping the details of FOG kinematics at a smaller time scale.

The results support a growing body of evidence that freezing of gait is precipitated by a centrally mediated high-frequency oscillation of the lower limbs that occurs prior to motion cessation and clinical onset. The results have practical implications for objective FOG monitoring [23, 26]; distal accelerometry has the potential to detect a FOG event prior to clinical onset, facilitating targeted intervention such as “on demand” sensory cueing.
